# Pleuroparenchymal Fibroelastosis: A Case Report

**DOI:** 10.7759/cureus.29211

**Published:** 2022-09-15

**Authors:** Oumayma Haloui, Fatima El Allam, Ouiame Nabou, Afaf Thouil, Hatim Kouismi

**Affiliations:** 1 Department of Respiratory Diseases, Mohammed VI University Hospital, Faculty of Medicine and Pharmacy, Mohammed First University, Oujda, MAR; 2 Department of Respiratory Diseases, Mohammed VI University Hospital, Oujda, MAR; 3 Research and Medical Sciences Laboratory (LRSM), Faculty of Medicine and Pharmacy, Mohammed First University, Oujda, MAR

**Keywords:** subpleural fibrosis, lung transplantation, lung biopsy, pleural thickening, pleuropulmonary elastosis, idiopathic interstitial pneumonia

## Abstract

Pleuroparenchymal fibroelastosis (PPFE) is a rare, idiopathic interstitial pneumonia. wherein the first symptom might be dyspnea or a dry cough. The condition can also be manifested with chest pain secondary to pneumothorax. While the definitive diagnosis is based on a histological evaluation (which is not often performed), a computed tomography scan shows findings, such as apical fibrosis and pleural thickening of the apical lobes, which help assess the diagnosis. We describe a case of PPFE diagnosed radiologically in a 69-year-old man. This case highlights that PPFE is a pathology that can go unnoticed for a long time, and patients might neglect the revealing symptoms such as coughing.

## Introduction

Pleuroparenchymal fibroelastosis (PPFE) is usually a rare, idiopathic disease characterized mainly by fibrosis in the upper lobes, subjacent parenchyma, and pleura. PPFE was first defined by Frankel et al. in 2004 [1]. Although the etiology of PPFE is unknown, the disease has been described to appear after lung transplantation, bone marrow transplantation, and chemotherapy [2]. It has also been linked to immune system disorders, recurrent respiratory tract infections, and family history [2]. We report a case of PPFE diagnosed radiologically in a 69-year-old man with a history of chronic smoking.

## Case presentation

A 69-year-old man reported concerns of a dry cough lasting for the past four years. He was a smoker (50 pack-year) before stopping the habit for the last two months. He also had chronic psoriasiform eczematous dermatosis evoking an angiodermatitis on skin biopsy three years prior to presentation, which was treated with topical treatments. The patient was admitted for the management of non-necrotizing dermo-hypodermitis in the dermatology department. On physical examination, we noted his oxygen saturation was 96%, his body mass index (BMI) was 17 kg/m^2^, and he had no thoracic deformity or clubbing. His pulmonary auscultation revealed no abnormalities. Examination of his lymph nodes revealed inguinal and retroauricular adenopathy. The patient also had scaly erythematous lesions on the forearms, knees, legs, back of the feet, palms of the hands bilaterally, genitals, and buttock area.

**Figure 1:**
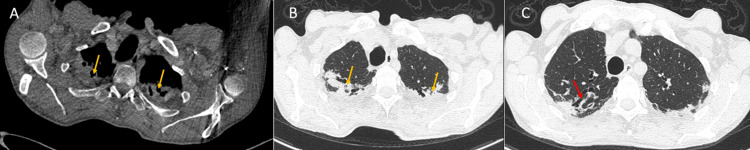
Coronal view of thoracic computed tomography with bone window study (A) and parenchymal window study (B and C) showing apical pleural thickening and subpleural consolidations (yellow arrows), and traction bronchiectasis (red arrow).

**Figure 2:**
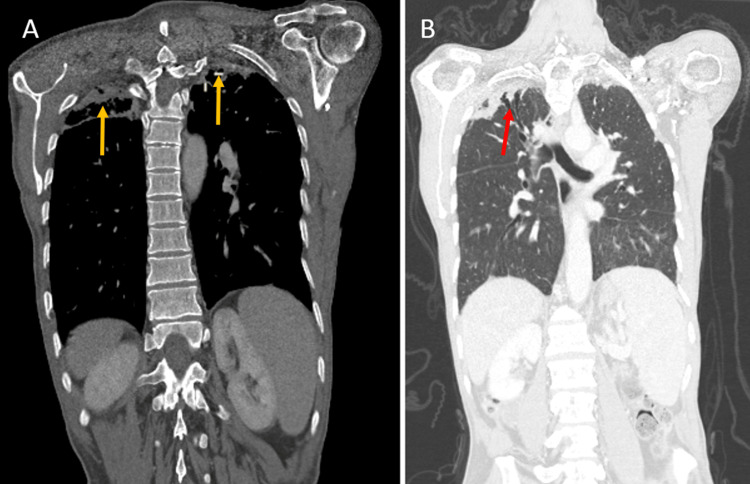
A: Axial view thoracic computed tomography showing apical pleural thickening and subpleural consolidations (yellow arrows). B: Axial view thoracic computed tomography showing traction bronchiectasis (red arrow).

The discovery of fibroelastosis was fortuitous in the computed tomography (CT) arterial portography (CTAP) obtained for the etiological diagnosis of the adenopathies and the erythematosquamous lesions (for suspected lymphoma). A chest CT showed bi-apical pleural thickening and subpleural fibrosis without lung volume loss. We also noted traction bronchiectasis (Figure 1 and Figure 2). A chest CT scan one year later showed stable findings in the bilateral upper lobe consistent with PPFE but no lesions consistent with malignancy in the chest or abdomen. His serologies were negative for human immunodeficiency virus and hepatitis B and C virus. His arterial blood gas test results were unremarkable. A lung biopsy for diagnosis was not recommended due to the high risks of the procedure including the possibility of non-return of the lung to the chest wall. The patient refused further treatment and rejected medical follow-up instructions.

## Discussion

Although the first description of PPFE in the Japanese literature dates back to the 1990s as "Amitani's disease" as well as other names like idiopathic pulmonary upper lobe fibrosis, it was mentioned for the first time under the term "idiopathic pleuroparenchymal fibroelastosis" by Frankel et al. in 2004 [1,3]. In 2013, PPFE was recognized as one of the two most rare idiopathic interstitial pneumonias (IIPs) along with idiopathic lymphoid interstitial pneumonia [4]. PPFE comprises 5.8% to 10.4% of idiopathic pulmonary fibrosis (IPF) cases [5]. Oda et al. found that 11 of 110 patients (10%) diagnosed with IPF had radiological PPFE while nine (8.2%) had the histological features of PPFE [6]. In a review of 445 patients with IPF, Lee et al. identified PPFE in 28 (6.3%) patients [7].

There is considerable variation in the age of presentation of PPFE; the idiopathic type generally occurs in patients of middle age, with a mean age from 20 to 80 years, with no sex predominance [2]. Frankel et al. suggested that PPFE might be associated with specific entities described as a "secondary form" [1]. The secondary forms of PPFE are most associated with autoimmune and connective tissue diseases, lung and hematopoietic cell transplantation, recurrent infections, chemotherapy agents, radiotherapy, chronic hypersensitivity pneumonitis, and familial (or hereditary) diseases, suggesting a possible genetic inheritance of the disease, among other forms. Furthermore, it appears that smoking is not considered to be related to the development of the disease [2,8].

Regarding the clinical symptoms of PPFE, the main symptoms are nonproductive cough and progressive exertional dyspnea. These symptoms can be insidious; pneumothorax can indicate PPFE by manifesting as chest pain [8]. Pneumothorax is one of the most common complications in idiopathic and non-idiopathic PPFE. PPFE patients have a higher incidence of pneumothorax than other IIPs [5]. Enomoto et al. reported that of 44 PPFE patients, eight (18%) had a previous episode of pneumothorax when first diagnosed [9]. Tanizawa et al. noted that 24 of 30 patients (80%) presenting a radiological PPFE had a previous episode of pneumothorax at the time of enrollment in lung transplantation [10].

Many patients with PPFE experience weight loss. Patients with PPFE showed a lower BMI than those with IPF [5]. Shioya et al. reported that an average BMI among 29 patients with radiological PPFE was 20.1 ± 3.25 kg/m^2^ [11]. Enomoto et al. reported that 44 patients with radiological PPFE had a median BMI of 17.2 kg/m2 [9].

The latency period of PPFE during which patients have no symptoms can be long, and abnormalities would only be noticed in radiological investigations. PPFE's clinical evolution can accelerate once symptoms appear [8]. A flattened rib cage (i.e., "flat chest," the ratio of the anteroposterior diameter of the thorax to the transverse diameter at the sixth thoracic vertebrae is less than in the general population) is an acquired and progressive deformation. It could be related to fibrosis and volume loss of the upper lobe. Basal crepitations are frequently heard on auscultation but are not as common as in other entities like IPF. Concerning pulmonary function, a restrictive outcome with decreased forced vital capacity (FVC), total lung capacity (TLC), and carbon monoxide diffusion capacity are the most common findings. In addition, residual volume (RV) may be within the reference range. Forced expiratory volume (FEV) during the first second and RV/TLC ratios may be modestly elevated because of compensatory hyperinflation in the basal lobes of the lung as a reaction to the fibrotic collapse of the superior lobes. The deterioration in lung function can lead to lethal respiratory failure, commonly associated with hypercapnia - more so in the later stages of the disease [2].

On CT scans of the thorax, PPFE patients typically have dense, irregular apical pleuroparenchymal thickening with moderate, associated, bilateral irregular nodular attenuation that increases with time. In the early stages of the disease, chest radiographs may show a slight bi-apical pleural thickening. As the illness evolves, other changes are visible such as pleuroparenchymal thickening, reduction in upper lobe volume, and superior retraction of the hila. Consistent axial and coronal reconstruction is recommended for the evaluation of PPFE cases. The typical CT pattern of PPFE was defined by Frankel et al. as a bilateral apical pleuroparenchymal fibrosis associated with subpleural lung involvement and loss of volume of the apical lobe volumes. However, these are also likely to appear in PPFE traction bronchiectasis, parenchymal retraction, and upward displacement of the hila. At the beginning stages of PPFE, CT scans reveal reticular and nodular opacities limited to the apexes of the lungs. With the progression of the disease, progressive pleuro-parenchymal thickening, septal thickening, reticulation, and traction bronchiectasis are noted. The bottom lobe involvement occurs with the reduction of volume and the lifting of the diaphragm. In the last stage of the disease, big cysts and bullae appear in the superior lobes [12]. Reddy et al. established diagnostic criteria for PPFE based on histologic and radiologic features (Table 1) [13].

**Table 1:** Criteria proposed for the diagnosis of PPFE CT: computed tomography; PPFE: pleuropulmonary fibroelastosis

Category	Histopathology	High-Resolution CT
Definitive [13]	Upper lobe pleural fibrosis with subjacent intraalveolar fibrosis accompanied with alveolar septal elastosis	Pleural thickening with associated subpleural fibrosis in the upper lobes without the involvement of the lower lobes
Consistent with PPFE [13]	Presence of intraalveolar fibrosis but 1) not accompanied by significant pleural fibrosis, 2) not subpleural predominance, or 3) not present in a biopsy of the upper lobe	Pleural upper-lobe thickening with associated subpleural fibrosis but 1) not distributed in the upper lobes or 2) with characteristics of a coexistent disease in other sites
Inconsistent with PPFE [13]	Absence of features of definitive and consistent diagnosis	Absence of features of definitive and consistent diagnosis

A biopsy might reveal benign results: dense pleural fibrosis, intra-alveolar fibrosis, and septal elastosis [12]. However, the biopsy is considered dangerous, as there is a high risk of the lung not returning to the chest wall after the procedure [3]. The prognosis is grim, and the decline in vital capacity is fast [3].

There is no effective pharmacological treatment available for PPFE. In some cases, aggressive treatment has been used with corticosteroids and immunosuppressants, but the evolution of the clinical course was progressive for many of these patients [2]. Furthermore, antifibrotics like pirfenidone and nintedanib have failed to modify the progression of the disease. However, some authors reported the success of reducing or stabilizing the decline in FVC [2]. Bilateral lung transplantation is currently the definitive treatment, with promising survival rates. Chronic respiratory failure must be managed with oxygen therapy, and proper infection control is important in treating PPFE as it is in other interstitial lung diseases [2].

## Conclusions

PPFE is becoming more common but its etiology remains unclear. While there is an improved understanding of the radiological and histopathological features, the prognosis is grim. There is still no effective treatment for PPFE. Our patient was diagnosed fortuitously via the CT neck, chest, abdomen, and pelvis scan as a search for neoplasia. This case highlights that PPFE is a pathology that can go unnoticed for a long time, and patients might neglect revealing symptoms such as coughing.
